# Supplementary figures and images for: Silencing of RpATG8 impairs the biogenesis of maternal autophagosomes in vitellogenic oocytes, but does not interrupt follicular atresia in the insect vector Rhodnius prolixus
Source: PLoS Negl Trop Dis. 2020 Jan 27;14(1):e0008012. doi: 10.1371/journal.pntd.0008012 (PMC7004382; doi:10.1371/journal.pntd.0008012)

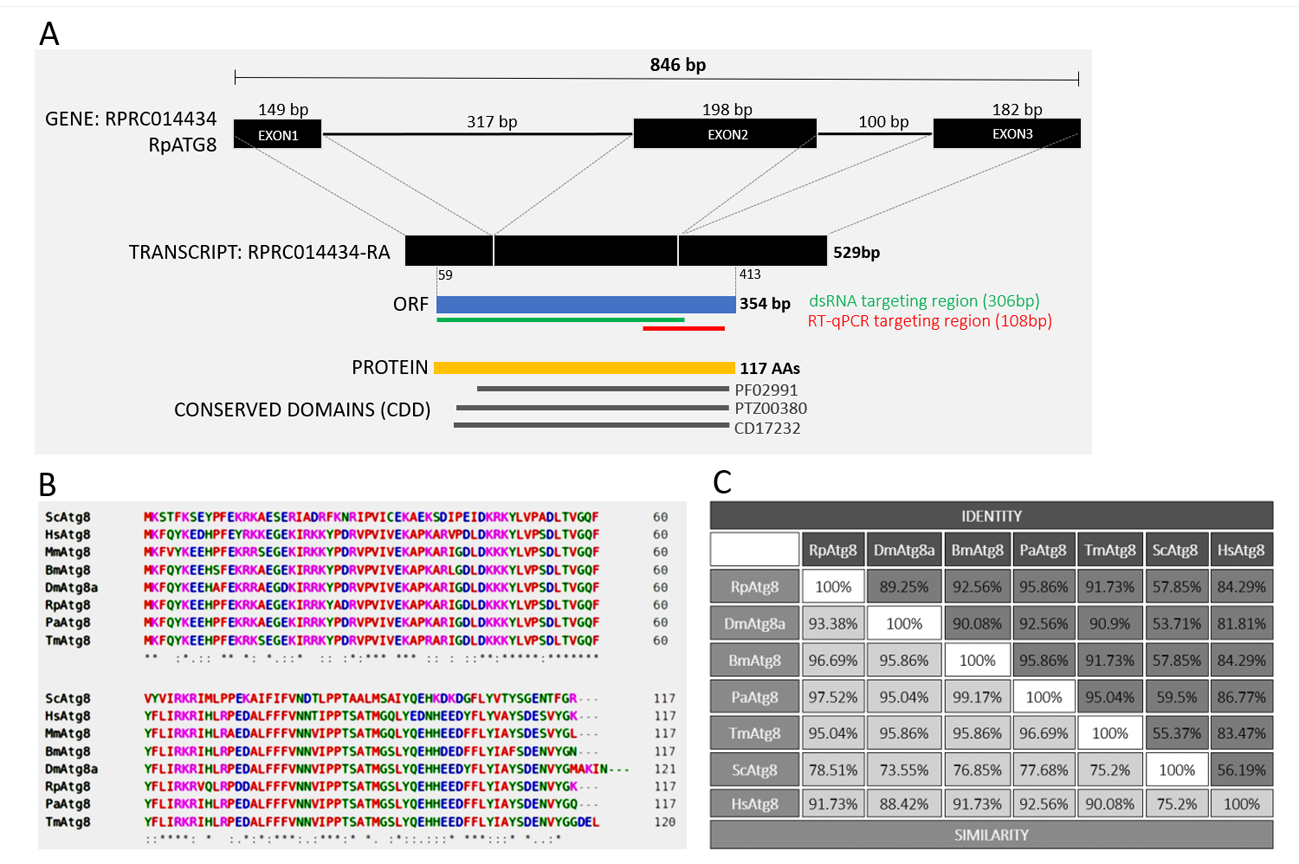

Supplement: S1 Fig — A. RpATG8 sequence analysis. Gene, transcript, ORF (with the primers targeting regions) and protein conserved domains are shown. Sequence information was obtained from Vector Base (https://www.vectorbase.org/). Conserved domains were obtained from the NCBI Conserved Domains Database. PF02991 (Autophagy protein Atg8 ubiquitin like); PTZ00380 (microtubule-associated protein); CD17232 (Ubl_ATG8_GABARAP). B. Multiple sequence alignment of ATG8 protein sequences of different species (Clustal Omega). C. Matrix of similarity and identity of ATG8 protein sequences from different species (SIAS Server). Reference sequences: Rp, Rhodnius prolixus; DmAtg8, Drosophila melanogaster (Gene ID 42132); HsAtg8, Homo sapiens (Gene ID: 11337); ScAtg8, Saccharomyces cerevisiae (Gene ID: 852200); PaAtg8, Periplaneta americana (CDS GenBank: AB856588.1); BmAtg8, Bombyx mori (Gene ID: 692938); TmAtg8, Tenebrio molitor (CDS GenBank: KM676434.1). (TIF) [file pntd.0008012.s001.tif]

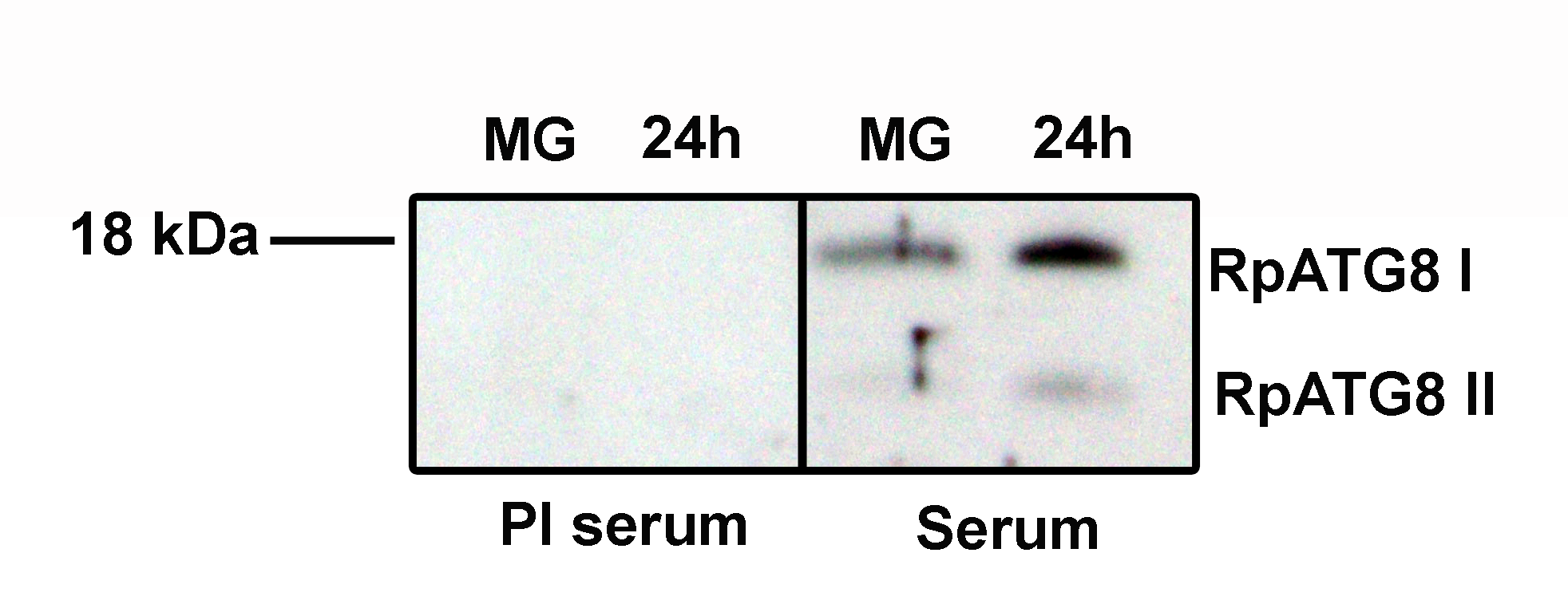

Supplement: S2 Fig — As controls, samples of the midgut and 24h-eggs were tested using the rabbit pre immune serum and RpATG8 immune serum. 45 μg of protein from each sample were used. The midgut was dissected 7 days after the blood meal. The eggs were homogenized in 50 mM HEPES, pH 7.4 20-24h after being laid by the females. The immunoblotting was performed as described in Methods. (TIF) [file pntd.0008012.s002.tif]
