# Supplementary material for: Silencing of RpATG8 impairs the biogenesis of maternal autophagosomes in vitellogenic oocytes, but does not interrupt follicular atresia in the insect vector Rhodnius prolixus
Source: PLoS Negl Trop Dis. 2020 Jan 27;14(1):e0008012. doi: 10.1371/journal.pntd.0008012 (PMC7004382; doi:10.1371/journal.pntd.0008012)
Supplement: Attachment — Submitted filename: Atresia_authors answers.docx [file pntd.0008012.s003.docx]

**Revised manuscript: #PNTD-D-19-01084**

**Author’s Answers**

Methods

Reviewer #1: The analyses in this manuscript aim to understand the role of the ATG8 protein and autophagic processes more generally in the process of follicular atresia in the Kissing bug Rhodnius prolixus. Overall the experiments are well designed, controlled and represent a comprehensive analysis of the role of autophagy and ATG8 in follicular atresia. The authors utilize a variety of approaches including qPCR, western blotting, RNAi, florescent microscopy, freeze fracture electron microscopy and measurement of physiological and fitness parameters to investigate the role of ATG8 and autophagy in atresia.
**Author’s Answer:** We thank the reviewer for the positive recommendation.

Reviewer #2: The authors characterized autophagy-related protein 8 in the insect Rhodnius prolixus, and studied the effects of silencing this gene on different physiological parameters. I found several methodological weaknesses in the work. Hence, many of the results could be misinterpreted.

-qRT-PCRs: According to the accepted standards, each qRT-PCR determination must use at least two housekeeping genes. See “Minimum Information Required for Publication of Quantitative Real-Time PCR Experiments (MIQE) Guidelines” (Bustin, S.A et al. Clin. Chem. 55, 611e622.).

**Author’s Answer:** Agreed. All RT-qPCRs were performed again using EF1 and 18S as housekeeping genes. The new data including the calculations using the two reference genes (18S and EF1) were included in the revised manuscript (Fig 1A, Fig 1B and Fig 3B). Calculations were performed using the geometric mean of the Cts from the two reference genes and expressed using the ddCT method, according to Vandesompele et al., 2002 as recommended by the MIQE guidelines. The methods were updated. The N was also increased for 6 biological replicates, as recommended by this reviewer on the other note.

For the organs (Fig 1A) and silencing experiments (Fig 3B) the overall patterns of expression levels were not altered with the new methodology. For the oogenesis experiment (Fig1B) the relative expression levels detected in the chorionated oocytes were markedly decreased when compared to the tropharium, previtellogenic and vitellogenic oocytes. This observation differs from than the data previously observed using actin as reference gene. We thank the reviewer for the suggestion/correction.

-For clarity reasons, primer sequence information should be presented as a table. For primer pairs used in qRT-PCR determinations, efficiency should be informed.

**Author’s Answer:** Done. Table 1 was added to the revised manuscript.

-RNAi mediated gene silencing: “using primers for RpATG6”, should be RpATG8.

**Author’s Answer:** Corrected.

- Detailed description of the injections is lacking: volume? method of injection? Etc. How many days after ecdysis did the females were injected? Virgin or mated females were used?

**Author’s Answer:** Done. The info added in the revised manuscript (methods>Insects) is copied here:

“Mated females are fed for the first time (as adult insects) in live-rabbit blood 14 to 21 days after the 5th instar nymph to adult ecdysis. After the first blood feeding, all adult insects in our insectarium are fed every 21 days. For all experiments, mated females of the second or third blood feeding were used”.

Sentence: “The bacterial MalE gene was used as a control dsRNA (ref?).” Reference is lacking. Also, please specify how MalE gene was amplified: primers, template, PCR cycling conditions.

**Author’s Answer:** The reference and info were added. The text in the revised manuscript (methods>RNAi silencing) is copied here:

“A fragment of 808 bp of the *E. coli MalE* gene (Gene ID: 948538) included in the control plasmid LITMUS 28iMal obtained from the HiScribe RNAi Transcription kit (New England BioLabs) was amplified by PCR using a T7 promoter-specific primer targeting the opposing T7 promoters of the vector. The cycling conditions were: 10 min at 95 °C, followed by 35 cycles of 30 s at 95 °C, 30 s at 52 °C and 60 s at 72 °C and a final extension of 15 min at 72 °C. The amplified fragment was used as a template for the synthesis of the control dsRNA (dsMal).”

Information on commercial sellers of consumables is incomplete throughout the manuscript. Importantly, off-target effects must be ruled-out with the use of another dsRNA fragment for all the determinations.

**Author’s Answer:** The commercial sellers were all added. Regarding the use of another dsRNA, we designed a dsRNA fragment targeting most of the RpATG8 ORF sequence (306bp of 354bp) and left a short 50pb sequence on the 3´ to design the reverse primer for RT-qPCR. So, there is no “room” to design a different dsRNA fragment. That being said, we did test *in silico* for potential off targets using our dsRNA fragment against the Rhodnius transcriptome database on Vector Base and found no significate results.

-Bioinformatics identification of RpATG8: this result is not described in methods. If authors identified the transcript they should specify the methodology used: which gene/s was/were used as query/queries? Which Blast strategy was implemented? Please provide detailed information.

**Author’s Answer:** Done. The info (copied bellow) was added to the revised manuscript (methods>gene identification).

**“Gene Identification:** The sequence of the RpATG8 transcript was identified from the R. prolixus digestive tract transcriptome database [30] (RP-1485, GAHY01001604.1) and then mapped to one single isoform of the gene RpATG8 (RPRC014434) corresponding to the transcript RPRC014434-RA in the Rhodnius genome assembly (Rpro C3), all available at Vector Base. The identification was accessed by similarity to the Drosophila melanogaster Atg8 sequence (NP727447.1) using tBlastn. Conserved domains were detected using the NCBI Conserved Domain Database (CDD). Alignments of the RpATG8 protein sequence to the ATG8 sequences from different species were performed using ClustalOmega”.

-Immunoblotting: Provide details on the secondary antibodies used and on ECL system employed.

**Author’s Answer:** Done. In Methods>immunoblotting. Revised text copied here:

“…Membranes were blocked in TBST (Tris 50mM, pH 7.2, NaCl 150mM, 0.1% Tween 20) containing 5% dry skimmed milk for 1h. Primary antibodies were diluted 1:2500 (RpAtg8, described above) or 1:10000 (Anti-alpha Tubulin, AbCam #ab7291) in the same buffer and incubated with the membranes for 14-16h (RpATG8) and for 1h (tubulin). The membranes were washed 3x for 5 min and then incubated with the secondary antibodies (Goat Anti-Rabbit IgG H&L HRP, AbCam #ab6721 for RpATG8 and Goat Anti-mouse IgG H&L HRP, AbCam #ab6789 for tubulin) diluted 1:3000 for 1h. After washing the membranes were developed using the Pierce™ ECL Western Blotting Substrate (ThermoFisher).”

-Most of the experiments have a very small sample size (n=3). This number should be increased in order to give confidence in the results.

**Author’s Answer:** Agreed. For some experiments we failed to mention the correct methodology and biological replicates (n) (we sincerely apologize for this neglect), for others, we performed new experiments to increase the n as suggested. The complete and correct descriptions of the biological replicates, pools, etc for each experiment were added to the methodology and/or legends of the revised manuscript. The corrections and new experiments are also described here:

- For the physiology experiments (digestion, survival, oviposition, hatching), we failed to mention in the methodology of our original manuscript that n=3 consists of 3 experiments, each of them including at least 8 females per treatment. Thus, the data includes at least 24 insects per experiment. This is now described in the revised manuscript. No new experiments were performed.

- For all RT-qPCRs the n was increased to 6 biological replicates (all including two endogenous controls, as suggested).

- For the experiments of macromolecule quantifications in the atretic oocytes, new experiments were performed: the Ns are now of protein (n=7), TAG (n=6) and polyP (n=4). For all experiments, each biological replicate was performed using a pool of 3 oocytes from 3 different insects.

Reviewer #3: In RNAi silencing methods chapter: “The bacterial MalE gene was used as a control dsRNA (ref?).” Is there a missing reference?

**Author’s Answer:** Yes, the reference was added, as well as the info regarding the amplification of the MalE gene. The text in the revised manuscript (methods>RNAi silencing) is copied here:

“A fragment of 808 bp of the *E. coli MalE* gene (Gene ID: 948538) included in the control plasmid LITMUS 28iMal obtained from the HiScribe RNAi Transcription kit (New England BioLabs) was amplified by PCR using a T7 promoter-specific primer targeting the opposing T7 promoters of the vector. The cycling conditions were: 10 min at 95 °C, followed by 35 cycles of 30 s at 95 °C, 30 s at 52 °C and 60 s at 72 °C and a final extension of 15 min at 72 °C. The amplified fragment was used as a template for the synthesis of the control dsRNA (dsMal).”

In Zymosan A challenge: “As controls, females were injected with 1 µl water alone.” You have been really injecting just water? No physiological solution?

**Author’s Answer:** Yes. Zymosan-A and dsRNAs are suspended in water and we chose to use the same vehicle to inject control animals. In our lab, we have previously performed tests of different vehicles for injections in *Rhodnius*. 1µl water injections have shown to maintain the overall levels of digestion, oviposition, F1 hatching rates and survival of adult females.

In Evaluation of survival and egg laying: “All groups injected with Zymosan-A, dsRNAs and controls” Specify if the group Zymosan/dsRNA is included and if control group was injected with dsRNA too.

**Author’s Answer:** Control groups were injected with Atg8 dsRNA too. The results are shown in Fig S1 (now Fig 6). No parental phenotypes are observed, only a slight decrease in the F1 hatching rates.

Not exactly clear what was meant by "Mortality and egg laying were recorded daily and weekly, "respectively"."

**Author’s Answer:** We meant that mortality was recorded daily and that egg laying was recorded weekly. The sentence was altered in the manuscript.

--------------------

Results

Reviewer #1: The results are clearly presented and explained and the figures are well designed and easy to interpret.

**Author’s Answer:** We thank the reviewer for the positive recommendation.

Reviewer #2: The results are correctlly presented, even though the methodological issues indicated above make that results difficult to interprete in many cases. I suggest to include an analysis of the sequence or RpATG8 identified (ORF, alignment with other species, conserved domains, intron-exons, etc) should be provided as supplementary information.

**Author’s Answer:** We thank the reviewer for the positive recommendation. As suggested, Figure S1 including the sequence analysis was added to the revised manuscript.

-The term “silenced” for the individuals belonging to the treated group is not accurate.

**Author’s Answer:** Agreed, the text was revised.

-“major organs of the adult female” is not a correct expression. The size of the organ is not related with physiological relevance. Please remove.

**Author’s Answer:** Agreed. As requested by one of the reviewers, additional organs were analyzed. The new data is now presented in Fig 1, and the term “major organs” was deleted from the description.

-I suggest to move figure S1 to the main body of the manuscript (not supplementary information).
**Author’s Answer:** Done. Figure S1 is now figure 6 in the revised manuscript.

Reviewer #3: By qPCR you showed that the ovaries express more RpATG8 than the midgut and fat body, the other two major organs of the adult female what I consider insufficient. You should offer the whole body organ tissues check. Would be more informative to show all other organs with possible autophagy activity too.

**Author’s Answer:** Agreed. We performed new experiments and included in the revised manuscript the expression levels of RpATG8 of all the organs that we could consistently dissect and extract RNA: fat body, anterior midgut, posterior midgut, ovary and flight muscle (from the thorax). Unfortunately, we couldn´t extract enough RNA (using a pool of 3 insects) from samples of the salivary glands and the crop. Please note that for all RT-qPCRs que N was increased to 6 and 18S and EF1 were used as reference genes, as suggested by reviewer #2.

“Type-2 atretic”, present a characteristic brown punctate pattern" is it not possible that this pattern could be caused by melanization?

**Author’s Answer:** This is an interesting point. For both groups of Zymosan-challenged insects (dsMal and dsAtg8) melanization was triggered in the hemolymph, as expected, and previously reported by Medeiros et al. 2009. However, the characteristic brown punctate pattern in the atretic oocytes was only present in Atg8 silenced females. Thus, we did not attribute this specific oocyte phenotype to the activation of melanization. This is now discussed in the revised manuscript (Discussion). The text is copied here:

“Furthermore, it is important to notice that because we used Zymosan-A to induce atresia, immune-response triggered melanization was observed in the hemolymph of both groups of Zymosan-challenged insects (dsMal and dsAtg8), as previously reported by Medeiros et al., 2009 [21]. However, the characteristic brown punctate pattern in the atretic oocytes was only present in Atg8 silenced females. Thus, we did not attribute this specific oocyte phenotype to the activation of the melanization cascade in the hemolymph.”

For Fig 3D the atretic type 1 is also similar to individuals treated with Zymosan A, if not could you provide the picture also for this treatment?

**Author’s Answer:** Yes, they look the same. The atretic oocyte type 1 shown in Figure 3D was dissected from a Zymosan-A dsMal insect.

For physiological and behavioral experiments do you have also results for just dsRNAi injections or they are all treated together with Zymosan A? If yes why was used combined treatment, or if you have results for just dsRNAi treatment incorporate in the charts.

**Author’s Answer:** We do have some physiological data for the dsAtg8 without Zymosan-A challenge, they are shown in Fig S1 (now Fig 6 in the revised manuscript). As observed, silencing of RpAtg8 in control females (without Zymosan-A) results only in a moderate decrease of the F1 hatching rates. No major changes were observed in the physiology of the silenced females, and no follicular atresia was triggered (as expected). Because our goal was to investigate the role of autophagy in the process of follicular atresia, a process known to allow adaptation to stress conditions, we performed the behavioral experiments only in challenged insects.

For locomotor activity you provided just one sentence: "Accordingly, the overall patterns of daily locomotor activity are also similarly decreased in challenged control and silenced females." Could you please provide more extensive description of Fig 5 results?

**Author’s Answer:** Sure.

“”.

Regarding the Fig 4E there is no explanation for this picture in whole text, so provide the description either in results section or discussion.

**Author’s Answer:** Figure 4E was mistakenly mentioned in the text as Figure 4F. Its description in “Results” was highlighted in the revised manuscript and copied here:

“Also, both types of atretic oocytes can trigger acidification of the yolk organelles during atresia, as seen by the fluorescence shift of acridine orange, a fluorescent marker of acid compartments (Fig 4E). Acidification of the yolk organelles, which culminates in the activation of yolk proteases, is characteristic of the follicular atresia degradation [37]”.

--------------------

Conclusions

Reviewer #1: The stated conclusions are supported by the data. I do feel that one of the more interesting findings of the analyses was placed in the supplemental materials (Figure S1). These results demonstrate a role for Atg8 and Autophagosome formation in embryonic development and egg viability. The majority of the presented results show that Atg8 is not required for the process of follicular atresia. This is informative, but I think the potential role of Atg8 in embryonic development is also worth highlighting in the context of its non-essential role in atresia. I think it would be worth considering moving Figure S1D into the main manuscript.

**Author’s Answer:** We thank the reviewer for the positive recommendation. Figure S1 is now Fig 6 in the main body of the manuscript.

There was one statement in the Discussion which I don't feel is accurate and should be modified or removed.
"In the context of insect vectors of human diseases, such as flies, bugs and mosquitoes, the ability of the hematophagous female to interrupt oogenesis and reallocate the energy stored in the oocytes is strategic for safeguarding vector capacity."

This somewhat implies that the reason for this process is to ensure that the bug maintains its vector competence. In reality this process is important for maintaining the bugs reproductive fitness by preserving deposited nutrients through resorption of partially developed oocytes. I don't believe maintenance of vector competence plays a role in the evolution of this process.

**Author’s Answer:** Agreed. Done.

Reviewer #2: The discussion of the data presented by the authors is clear and complete.

**Author’s Answer:** We thank the reviewer for the positive recommendation.

Reviewer #3: Article conclusions are supported by the presented data and discussion includes the contribution to studied topic.

**Author’s Answer:** We thank the reviewer for the positive recommendation.

--------------------

Editorial and Data Presentation Modifications?

Reviewer #1: I would suggest moving figure S1D to the main text and to modify the statement regarding the role of atresia in maintaining vector competence.

**Author’s Answer:** Done.

Reviewer #2: I suggest English edition of the manuscript.

**Author’s Answer:** Done.

Reviewer #3: Minor Revisions are needed according the previous comments and completion of experiment showing all tissue expression of ATG8 gene is essential.

**Author’s Answer:** We thank the reviewer for the positive recommendation. The experiment including other organs/tissues expression was performed and included. Please note that it was not possible to dissect and extract RNA from ALL the organs of the insect. We included the organs that we could reliably dissect and prepare the samples.

--------------------

Summary and General Comments

Reviewer #1: Overall, I feel this is a solid and well written paper which just requires some minor modifications prior to publication. While the results are not earth shattering, they provide some interesting insights into the relationship between autophagy and follicular atresia in an important vector species.

**Author’s Answer:** We thank the reviewer for the positive recommendation.

Reviewer #2: The manuscript address a relevant subject, and could be interesting for publication. However, the experimental and methodological problems referred above must be addressed.

**Author’s Answer:** We thank the reviewer for the positive recommendation. We agree and addressed the methodological issues as suggested.

Reviewer #3: (No Response)

To all reviewers: Please note that, as requested by the Plos Data Policy, the data: “As a control, we tested the immune serum in samples of a somatic tissue, the midgut epithelium, dissected from vitellogenic females, and found that it labeled only the free form of RpATG8 (ATG8-I), as expected, given that autophagy is usually triggered under nutritional stress”, not referenced before (as data not shown), is now described in Figure S2 of the revised manuscript.
