# Supplementary material for: Silencing of RpATG8 impairs the biogenesis of maternal autophagosomes in vitellogenic oocytes, but does not interrupt follicular atresia in the insect vector Rhodnius prolixus
Source: PLoS Negl Trop Dis. 2020 Jan 27;14(1):e0008012. doi: 10.1371/journal.pntd.0008012 (PMC7004382; doi:10.1371/journal.pntd.0008012)
Supplement: Attachment — Submitted filename: Atresia_authors answers R2.docx [file pntd.0008012.s004.docx]

**Revised manuscript: #PNTD-D-19-01084**

**Author’s Answers R2**

The manuscript is greatly improved, but there are still a few minor comments to address.  The reviewers have also suggested that the manuscript should be edited for grammar and style.

**Author’s Answer:** The minor comments are addressed bellow and a grammar and style revision of the manuscript was performed by a native speaker colleague.

**Reviewer's Responses to Questions**

**Methods
Reviewer #1:** (No Response)

**Reviewer #2:** Authors addressed the most of the Reviewer comments. I suggest minor changes:
When Vector Base is cited, include the Internet address of Vector Base.

**Author’s Answer:** Done.

How many days after injections the insects were fed?

**Author’s Answer:** Six days after the dsRNA injections. This info was highlighted (in red) in *Methods>RNAi silencing* in the revised manuscript.

Please clarify that off target effects cannot be rouled out given the reasons exposed by authors in the Reviewer responses.

**Author’s Answer:** Done. The clarification was added to the discussion in the revised manuscript (in red) as exposed in our previous response. Text copied bellow:

“Additionally, it is important to mention that our dsRNA fragment targets most of the RpATG8 ORF sequence (306bp of 354bp), so there is no extra room in the sequence to design an additional dsRNA for a different region. Therefore, although we tested *in silico* for potential off targets with no significant predictions, it is not possible to completely rule out off target effects”

**Results
Reviewer #1:** (No Response)

**Reviewer #2:** The results are clear and completelly presented. The information is original and relevant.
**Author’s Answer:** We thank the reviewer for the positive recommendation.

**Conclusions
Reviewer #1:** (No Response)

**Reviewer #2:** The results are well discussed in this section.

**Author’s Answer:** We thank the reviewer for the positive recommendation.

**Editorial and Data Presentation Modifications?**

Use this section for editorial suggestions as well as relatively minor modifications of existing data that would enhance clarity. If the only modifications needed are minor and/or editorial, you may wish to recommend “Minor Revision” or “Accept”.

**Reviewer #1:** (No Response)

**Reviewer #2:** I suggest English edition.

**Author’s Answer:** The text was revised by a native speaker colleague. The changes are shown in the tracked changes copy.

**Summary and General Comments:**

**Reviewer #1:** The authors have adequately addressed my concerns and those of the other reviewers. I feel that the paper is appropriate for publication in PLoS NTDs.

**Author’s Answer:** We thank the reviewer for the thorough revision and for the positive recommendation.

**Reviewer #2:** The authors addressed the Reviewers suggestions. The manuscript has been improved respect to the original version. After minor revision, I consider that the manuscript is suitable for publication in PLoS NTD.

**Author’s Answer:** We thank the reviewer for the thorough revision and for the positive recommendation.
